# Health Capital and its Significance for Health Justice

**DOI:** 10.1093/phe/phaf001

**Published:** 2025-02-08

**Authors:** Ben Davies, Thomas Schramme

**Affiliations:** Department of Philosophy, University of Sheffield, Sheffield, UK; Department of Philosophy, University of Liverpool, Liverpool, UK

## Abstract

This paper outlines a novel framing of the normative significance of health by considering the idea of ‘health capital’. Health capital is a set of health-related assets of individuals that enable them to pursue their interests and to collaborate with others. The specific contribution of this paper is to establish the notion of health capital beyond a metaphorical idea and to initially explore the repercussions of it for theories of health justice. We propose a sufficientarian approach to health capital justice. Our theory claims that social justice requires enabling people to achieve enough health capital to meet threats to health. This is a dynamic ideal, establishing claims of justice over time. The overarching goal is to avoid disadvantageous tipping points of health depletion.

A banner at the sports hall of the University of Liverpool states: ‘Health is your wealth. Spend it wisely’. This is an interesting way of looking at health—as something that comes in a certain amount (‘wealth’), as something that can be used (‘spend’), and as something that can be influenced by reason (‘wisely’). We might see the view contained within this admonishment as seeing health as a kind of capital: health capital.

The fact that health is a form of wealth has perhaps never been as obvious as today. Indeed, citizens increasingly tend to perceive human health from an economic point of view, especially in individualistic capitalist societies. For instance, many people check their ‘investments’ on activity trackers and compare these achievements to other people’s efforts. Relevant policies additionally bolster and encourage such a perspective. During the coronavirus disease 2019 pandemic, a spotlight was put on health as an individually and socially enabling factor. We do not need to approve of these developments that can be witnessed in many modern societies, but they are hard to ignore, and they have significant repercussions on many people’s lives.

Health capital is a set of health-related assets of individuals that enable them to maintain their health and thereby be able to pursue their interests, relate to and collaborate with others, and gain competitive advantages. It comprises, firstly, a specific malleable health-related constitution of a person, which we call health stock, and, secondly, means of health production. Other things being equal, the more health capital people have, the easier it will be for them to live their life according to their own ideals. Since the goods created by health capital, for instance, a specific level of fitness, are themselves constituents of further health capital, there can be both vicious and virtuous circles of health capital changes. Those who are rich in health capital are in a good position to create more health capital, whereas those who are poor in health capital have an increased vulnerability to further health capital losses. Our paper is a first exploration of the overarching research question as to what is due to people as a matter of social justice to prevent vicious circles of health depletion. The specific contribution of this paper is to establish the notion of health capital beyond the metaphorical idea contained in the University of Liverpool’s banner and to initially explore the repercussions of this idea for theories of health justice.

We will emphasise the advantages of our novel framework to assess social conditions and public policies in terms of social justice. We broaden the horizon from health justice, which has dominated the discussion for some decades, to health capital justice. Related and similar frameworks have been proposed in the literature (e.g. Gee and Payne-Sturges, 2004; Prah Ruger, 2010; Venkatapuram, 2011; Tengland, 2016; Reynolds, 2021), and we do not set ourselves in opposition to them. Still, we believe that our model offers advantages, especially in terms of conceptual rigour and transdisciplinary amenability.

In the first section, we clarify the crucial notion of health for our purposes. We understand health not simply as the absence of disease but as an individual asset that comes in grades. From this perspective, health is conceptualised as a disposition that makes it more or less likely to prevent and fight disease. Both organismic constitutions and circumstantial determinants of health contribute to this gradual disposition. It is, however, important not to confuse the evaluative and normative significance of a minimal concept of health—the absence of disease—and a positive conception over and above the absence of disease. Being less healthy does not have the same relevance as being unhealthy from the perspective of social justice.

In the second section, we focus on the concept of health capital. This is a broad notion that includes elements that are intrinsic and extrinsic to persons. It also enables us to theorise relational aspects, for instance, potential hazards or benefits to a person’s health represented by social environments and interpersonal relationships. In the third section of the paper, we accordingly discuss the foundations of a theory of health capital justice, whose details will however have to be laid out elsewhere. We propose a version of a sufficientarian theory of justice. Our theory claims that social justice requires enabling people to achieve enough health capital to meet threats to health. This is a dynamic ideal, establishing claims of justice over time (Valles, 2018, 57ff.). The overarching goal is to avoid disadvantageous tipping points. We provide arguments for sufficientarian health capital justice. In the fourth section, we defend our view against objections, specifically from the point of view of egalitarian theories of health justice. The fifth and final section summarises the main points of the new normative framework of health capital justice, which are presented in this article for the first time.

## The Concept of Health and its Normative Significance

Social justice, when concerned with human health, had for a long time been understood to concern access to health care provision in situations of medical need (e.g. Daniels, 1985). Public health research—which produces findings about the health of populations—has additionally led to the acknowledgement of further, especially social, determinants of health. Consequently, health justice is currently understood to include far more than just medical provision; for instance, it incorporates meaningful work opportunities or access to recreational spaces (Daniels, 2008; Venkatapuram, 2011; Schramme, 2019). In other words, maintenance of health, not merely returning to health in cases of illness, has come into the focus of social justice discussions. This is an important development because social conditions that were not necessarily seen as raising concerns of justice have been acknowledged via the normative concern with human health. Health differences entered the political debate and have since been widely deemed unjust, at least if they are preventable (e.g. Marmot, 2004; Deaton, 2013). However, the relatively recent conceptualisations of health justice have important weaknesses, which riddle the current state of academic and political discussion on health justice. First, a conceptual confusion regarding the notion of health; second, an evaluative confusion concerning the value of absence of disease in distinction to the value of conditions maintaining health.

Health, from a common medical perspective, is interpreted as the absence of disease or dysfunction. For instance, from the point of view of a primary care physician who examines a concerned person in their surgery, the important point is whether the patient has a disease. In terms of health, everyone who has no disease is equal from this point of view. Accordingly, this perspective works with an absolute concept of health; it is not gradable. It is true, of course, that medical practice might call for judgements about grades of severity of diseases. But this does not introduce grades of health. Rather, health is here defined in negative terms, as absence of disease.

For specific purposes of medicine, people can also be placed along a spectrum of health. From this perspective, health is not restricted to the absence of disease (WHO, 1948; Nordenfelt, 1987; Schramme, 2023). Rather, it addresses conditions of people over and above the absence of disease, specifically their disposition to prevent disease. In this sense, some people might be healthier than others (Schroeder, 2013), say, because they exercise regularly, live in a supportive relationship or because they are confident in accessing medical support. In contrast to the negative interpretation of health as an absence of disease, placing health on a spectrum enables comparisons and rankings of health statuses between individuals. Grades of health are described in positive terms, that is, in respect to the presence of something, for instance a certain level of fitness. However, the fact that a person is less healthy in this sense, for instance due to a higher propensity to fall ill because of specific social circumstances, does not mean that this person is ill. Briefly put, being less healthy is not the same as being unhealthy. It is important to keep separate these two types of health—a positive concept and a negative concept—to avoid confusion (Tengland, 2010).

Conceptual confusion can be specifically harmful for purposes of social justice because being less healthy does not carry the same normative urgency as being unhealthy. Disease usually has normative significance because it indicates a need, which will result in harm if not fulfilled. In contrast, lack of a comparatively worse state of positive health might indicate a disadvantage, but not a need. So, the mentioned conceptual distinction helps to avoid the mentioned evaluative confusion. We must not confuse the direct normative significance of ill health with the indirect and much more contested normative significance of disadvantageous health dispositions.

To add another layer that makes the conceptual landscape even more confusing, the modern debate on health justice often refers to populations, mainly along social strata. For instance, it is stated that ‘the lower a person’s social position, the worse his or her health’ (Marmot, 2010, 15). Such claims do not refer, of course, to every individual, but to populations; they concern statistical correlations. In other words, population health is not the same concept as individual health (Arah, 2009; Schramme, 2017). The health of populations is correlated to social conditions, such as the level of education, employment, quality of housing and of the natural environment. In this respect, we need to again carefully disentangle the conceptual intricacies. Accordingly, we should distinguish between organismic health, which refers to the constitution of an individual, and the social conditions of health maintenance. A person might be in good health due to a certain level of organismic fitness or due to a social environment that is amenable to prevent diseases.

Although we do endorse a positive notion of health, similar to scholars who support a capability to be healthy (Venkatapuram, 2011) or health capabilities (Prah Ruger, 2010), our approach differs in relevant respects. As explained, we firstly insist on a conceptual distinction between health as an absence of disease and health as a disposition. Importantly, our normative framework is closely linked to the traditional aim of health justice, which consists in treating and preventing disease. So, the negative notion of health provides normativity, whereas the focus on positive health adds a layer of conditions and circumstances that support health maintenance and hence establish further potential concerns of justice. Secondly, we distinguish between organismic conditions and social conditions, despite the fact that they both contribute to the maintenance of health.

Capabilities represent people’s freedoms to achieve various ‘beings and doings’, as Amartya Sen put it a while ago (Sen, 1992, 39ff.). So, there is already a combination of organismic conditions and circumstances within the notion of capabilities. This does not as such pose a problem, but it is detrimental to theories that use capabilities within the context of health justice. If we conceive health itself as a meta-capability or, alternatively, social conditions as health capabilities, then the circumstances that contribute to health are placed in the same category as the health constitution of a person (Tengland, 2020). In consequence, if health is seen as a capability or in relation to capabilities, and capabilities are interpreted as the relevant metric of social justice, then having less health capabilities is automatically identified as a potential injustice. But this move short-circuits the significance of organismic ill health with comparatively worse health-related circumstances.

For instance, Jennifer Prah Ruger claims: ‘Deprivations in people’s health capability are unjust because they unnecessarily reduce the capability for health functioning and the exercise of agency, and undermine human flourishing’ (Prah Ruger, 2009, 266). But this is too quick. To have less health capabilities does not mean to be unhealthy. A smaller endowment of health capabilities as such has no direct normative significance, unless it is connected to the presence of illness. The assumed normative urgency of impaired health capabilities cannot automatically be drawn from the badness of ill health, because the relevant situations people with less health capabilities face—relatively fewer health capabilities as opposed to disease—are not the same. Briefly, health as a positive notion should not directly be used as a metric of social justice.

In fairness, Prah Ruger refers to ‘deprivations’, and this term can be read as referring to a threshold of enough capabilities (Prah Ruger, 2009, 266, 269), so that the unjust situation is meant to describe a situation of health-related need, which will result in harm if not met. Seen in this way, her health-related capabilities approach resembles our proposed sufficientarian theory. Yet, as we have argued, it is nevertheless a shortcoming to combine in one category the instruments of health maintenance and the organismic conditions themselves. This leads too quickly from the plausible claim that social justice requires enabling people to be healthy to the further claim that health capabilities have ‘special moral importance’ (Prah Ruger, 2016, 279).^1^

We submit that our framework, which we further develop in the following section, is more plausible because it does not fuse concerns regarding either organismic conditions or social circumstances. Furthermore, since deprivations of positive health are not in themselves unjust, deprivations in health capabilities are not themselves unjust, either. So, we need another step in an account of health justice to determine which impairments of health-related ‘beings and doings’ are unjust. The extra step is indeed acknowledged by public health researchers. For instance, Margaret Whitehead’s influential definition of health inequity ‘refers to differences in health which are not only unnecessary and avoidable but, in addition, are considered unfair and unjust’ (Whitehead, 1991, 220). The injustice of health-related differences has to be established separately and must not be based on the normative urgency of ill health. We believe that the framework of health capital will help to get a better grip at health-related injustices.

In summary, in this section, we have claimed that negative health—the absence of disease—directly carries normative urgency, whereas positive health does not. Still, the significance of organismic health dispositions and of other means of health maintenance make them pertinent elements of theories of health justice. Therefore, positive health becomes a secondary concern of health justice. In the next section, we lay out the framework of health capital to combine the purposes of conceptual and evaluative precision.

## The Concept of Health Capital

It is important to appreciate that the term *capital* as such does not carry any necessary relationship to capitalism (although it did in Marx’s conceptualisation). We use *capital* as an analytical concept to refer to ‘actually usable resources and powers’ (Bourdieu, 1984, 108). It is a fitting category because health can be achieved with the support of means of production and forms of labour. We hence describe health (although not merely) as an asset, in congruence with factual developments in many societies. Such phrasing might easily be misinterpreted as an endorsement of the commodification of health, which is however obviously not intended by our framework. Indeed, health capital can be used as a critical category. Specific social realities can be identified as problematic, because they impair access to means of health production for some people. For instance, if family support for children is undermined due to economic pressure on parents to maintain multiple jobs, then these children’s health capital is reduced, potentially to a degree that is intolerable from a perspective of social justice.

The concept of health capital is not new. It was introduced in the discipline of health economics by Michael Grossman (1972). However, Grossman had different purposes from ours. He was interested in explaining changes in the demand for the commodity ‘good health’, in other words, to explain certain consumer behaviour. The notion of health capital has not gained a foothold in the relevant philosophical debates, despite its influence in health economics.

Grossman himself did not analyse the term *health capital*. After all, his main concern was not conceptual analysis but to devise a model that served the purpose of predicting demand for health, including mainly medical care. He stated that ‘(t)he central proposition of the model is that health can be viewed as a durable capital stock that produces an output of healthy time. It is assumed that individuals inherit an initial stock of health that depreciates with age and can be increased by investment’ (Grossman, 1972, 223). In other words, health is seen as if it was a form of capital that can—at least up to a point—be produced, consumed and maintained (Zweifel *et al*., 2009, 75ff.). This is an important insight, although objections have been raised towards Grossman’s assumption of an inherited stock of health that depreciates over time. An alternative model, the health-deficit model (Dalgaard and Strulik, 2015) starts from biological findings regarding the process of senescence. It agrees with Grossman that health and thereby the age of death can be partly determined by health investments. In contrast to the idea of a depreciation of health stock, however, this model sees health investments as means of slowing the biological process of ageing. For the purposes of our paper, the quarrel between the two economic models of health capital is irrelevant, because we use a much broader notion of health stock and health capital, and we do not aim at the prediction of individual choices.

The quest to achieve a tighter definition of health capital can start by taking a closer look at the possible functions of the word ‘health’ within the expression. First, health itself, the organismic constitution of an individual, can be deemed a capital of a person. Let’s call this health stock, following Grossman. Second, the capital of a person, which is not itself a constitution of the person, can be seen as relevant for health. In this second sense, we might want to use the expression ‘health-related capital’ or ‘capital for health’. Within a conceptualisation of health as a scalar, there is no problem in allowing assets that are exogenous to the person to be included. After all, enabling conditions of health have an impact on the level of individual health, understood as a positive notion, over and above the absence of disease. People who live in advantageous social circumstances are healthier than others in this respect, because they have a better health disposition, that is, they are less likely to fall ill. Let’s call this second form of health capital accordingly the means of health production.

The health stock of an individual is initially constituted of a natural and social endowment. Whether a person has a genetic disease, or a precarious disposition, is partly determined by natural causes but also by acquired skills and competencies. One’s health stock comes with direct gratification—not to be ill is itself a form of concealed wellbeing (Gadamer, 1996). Being healthy is accordingly a direct constituent of being well, even if it is itself not usually experienced as such. Health can also be used as a means to reach other goals that are constitutive of one’s wellbeing, such as performing meaningful activities. Finally, one’s health stock can be used in the production of relational assets, for instance competitive advantages on the labour market.

The second element of health capital, the means of production of health, is multifarious. Endogenous health, i.e. one’s level of individual health, can itself be used to achieve more health. A fit person will typically find it easier to stay fit and perhaps get even fitter, whereas a chronically ill person will often develop further illnesses. This means that health stock and means of production of health are related, indeed often causally connected. We will later highlight the normative significance of this circularity, which can take a virtuous and a vicious form—from health to more health and from a bad health disposition to even worse health.

Exogenous factors, such as accessible health care provision, clean environment and beneficial social conditions, are also important means in the production of health. Social and cultural factors, for instance the publicly available level of knowledge about medical conditions and possible treatment options, can also be mentioned. In Grossman’s original model, the social determinants of health, which feature so important in the public health literature, were not fully visible (Gilleskie, 2008, 62; Galama & Kippersluis, 2013, 277). These days, they are among the most researched drivers of health, and from a perspective of justice, they have also entered the spotlight (Wilkinson, 1996; Venkatapuram and Marmot, 2014). Still other factors of this form of health capital are individually allocated, but culturally determined, such as the level of one’s ability and confidence to engage and communicate with health care providers (cf. Shim, 2010). We believe that including all these diverse means of production of health under the umbrella of health capital allows new perspectives and more nuanced assessments of health justice.

**Figure 1.**
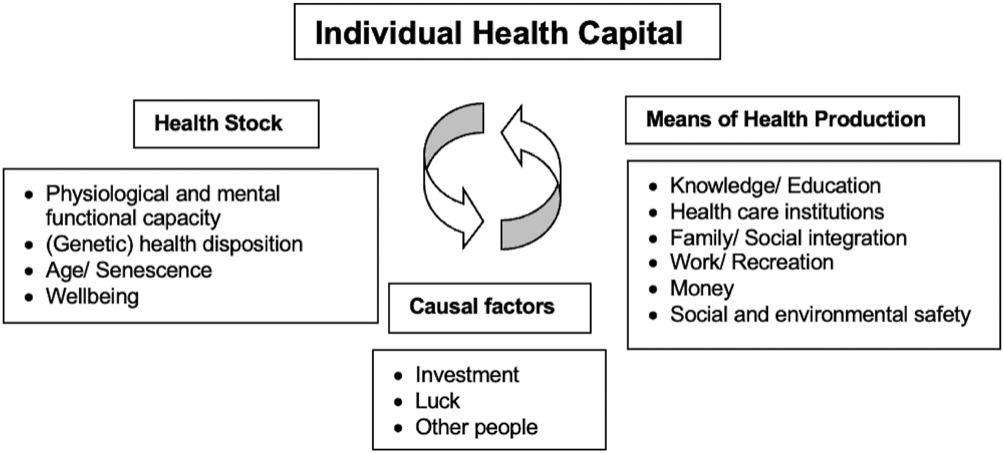
Individual health capital and its components. Health capital is a set of health-related assets of individuals that enable them to maintain their health over time. Health capital comprises health stock and means of health production. Health stock is the organismic constitution of a person, including biological and mental states or dispositions. Means of health production include health-relevant external resources available to an individual. Both sets of health-related assets interact in positive and negative ways (as indicated by the curved arrows), for instance, lack of family support might increase the negative impact of senescence on an individual. The level of health capital is causally influenced by numerous factors, such as personal and public investment (e.g. regular exercises or vaccination programmes), (bad) luck (e.g. accidents) and the impact of other people’s behaviour (e.g. violence).

Our twofold conceptualisation of health capital, including health stock and means of health production, is in line with recent publications from different disciplinary sources (Arrow *et al*., 2014; Schneider-Kamp, 2021).^2^ It also has potential connections to established theories of human, social and cultural capital (Bourdieu, 1986; Wilkinson, 1996). In summary (Figure 1), we understand individual health capital as the available assets which constitute a person’s level of positive health, and which enable them to influence their health status with a specific probability over time.

Importantly, health capital can be affected by others. The relational aspects of health capital have been discussed, albeit in different terms, in the philosophical literature on social justice. Individual health can be described as a positional good (Brighouse and Swift, 2006; Davies, 2022). The value of our health is partly determined by the health status of others. If a person has only a mild chronic illness, such as diabetes, her health capital might still be relatively large in this respect. However, if she happens to live in a society of extremely healthy people, she might nevertheless be at the lower end of the distribution of individual health capital. This shows how one’s position within society can affect the value of one’s assets. After all, the relative position of a person within a distribution of goods determines one’s chances of success, especially in competitive scenarios. For example, if a child needs to spend a couple of hours per day tending to obsessive-compulsive behaviour, they will have less time to spend on other learning activities.

A significant benefit of our distinction between health stock and means of health production is to keep the organismic condition of health separate from the instruments that can be used to produce health. This is vital because the two elements raise different normative concerns. Admittedly, the difference between health stock and means of health production is not rigid. Indeed, as mentioned earlier, a good health condition is itself useful when investing further into health. Nevertheless, the distinction is important, because in the relevant literature on the social determinants of health, it is sometimes difficult to maintain a separation of inequalities in health as opposed to inequalities regarding the means of health production. A person who has a low degree of, say, knowledge regarding healthy nutrition does not thereby have a poor health condition, or even a form of illness. Altogether, the twofold notion of health capital enables us to include insights from health economics regarding health production and from social epidemiology regarding the determinants of health.

## Sufficientarian Health Capital Justice

Although the interconnectedness of health and other goods is not a new insight, the idea of health capital provides an original framework through which to view these interactions. This framework might be called ‘health-focussed’: interactions between different elements of individual lives, and between different individuals, are framed in terms of what produces health, and what health produces (see also the WHO’s (2014) ‘health in all policies’ approach). We think this is a valuable conceptual tool. While it is not the only way to view a network of goods and relationships between them, it can be helpful to adopt a framing in terms of a widely accepted value. Those who think that significant health inequalities are especially wrongful (Daniels, 2008; Riddle, 2014; Badano, 2016; Rumbold, 2021; Horton, 2022) but are willing to accept significant inequalities in other parts of our social world, may be challenged by a health-focussed framing that notes the interconnectedness of health with other goods.

Within our sufficientarian conception, the aim of social justice is to provide enough health capital to balance the health-related threats people face. Our framework has clear parallels with Wolff and De Shalit’s (2007: Chapter 7) ideas of ‘fertile functionings’ and ‘corrosive disadvantage’ (see also Goodin, 2023). As Riddle (2014: 75–77) suggests, ill health is often a corrosive disadvantage, making one more vulnerable to further disadvantages. We would go further: since ill health is often corrosive, decisions that lead to significant depletion of health capital are also often corrosive. With that in mind, what might a ‘health-focussed’ framing of health capital do for our thinking about justice? We do not aim to outline a full theory of health capital justice in this paper, but rather to point towards a couple of ways that the idea of health capital may shape our thinking about social justice.

In our view, health inequalities in societies are not themselves unjust, although they are often indicators of injustice. They are potential symptoms or signals of a social pathology (Ashcroft, 2013; Preda, 2018). For instance, the fact that certain health conditions follow a social gradient can bear evidence of social ills, such as health illiteracy (Kickbusch, 2001), suffered by specific socio-economic groups. We therefore believe that many concerns of egalitarian thinking and of the social science literature focussing on the notion of inequity can be integrated into our sufficientarian theory of health capital justice. Inequities are usually due to insufficiencies in specific respects.

Our positive argument for a sufficiency approach to health capital begins from the value of choice (Scanlon, 1986, 2024). As we have suggested, a central point lying behind the concept of health capital is the idea that health is something that one can invest in, and which can itself be invested in other goods. Of course, investments in an individual’s health are not always done by individuals themselves; public health measures are one way for state institutions to invest in the health capital of many individuals at once. Yet, state institutions should not insist that individuals always prioritise individual health, since people value goods other than health. Things are a little more complex when it comes to health capital. There can be time-based trade-offs in health capital; some may prefer to spend health capital now and risk having less in the future, whereas others may prefer to save. Thus, ‘maximising’ may mean different things to different people, and to the same person at different times. But it is also true that one need not rationally maximise health capital, even though it is a broader idea than health. For instance, some may wish to take enjoyable risks that do not promote their health capital at all.

We think that all else being equal, it is valuable for people to decide for themselves how to manage their health capital investment. This suggests an initial, low threshold for health capital justice: that people have sufficient health capital *to be able to decide* reasonably how to manage their health capital investments. This involves familiar sufficientarian values such as self-determination (Shields, 2016), but also sufficient external opportunity to make a range of investment decisions. This capacity is of sufficient importance that even if people make health capital investment decisions that will undermine it, they should generally be protected from the most serious effects of health risk. This, of course, implies traditional aims of health care justice, that is, some level of provision for treatment of occurrent diseases.

The iterative, dynamic aspect of health capital does suggest the potential for a somewhat more demanding threshold, on top of the minimal protection for enabling people to make decisions about their health capital (this would result in a ‘multi-level’ approach (Huseby, 2020)). We are unsure whether this additional, higher threshold is appropriate, given that it implies greater intervention in people’s health capital investment decisions. Such a threshold is more complex than the bare capacity for making investment decisions, but it may both be more cost-effective (requiring fewer interventions overall) and fairer given that people begin from very different starting points. For instance, what constitutes a sufficient level of health-related knowledge will depend in part on one’s health stock; someone with lower levels of health stock may need greater knowledge of how to maintain and build on what they do have than someone with higher initial stock. Nevertheless, our approach would, we think, still leave people with plenty of freedom of how to invest their health capital.

Given the prospect of both positive and negative cycles that people can become embroiled in, an alternative way to fix a sufficientarian threshold is to think about the possible tipping point(s) between an individual being drawn into a depletion cycle (where initial decisions that deplete health capital make future investment decisions much more difficult, and so tend to further depletion) or being able to enter into an accumulation cycle (where initial decisions that increase health capital make it significantly easier to make future investment decisions, and so tend to further accumulation). The idea of tipping points is not supposed to replace all other considerations of what constitutes enough health capital, but rather facilitates highlighting specific, crucial health threats that ought to be met; or, in the case of accumulation cycles, which ought to be sought.

Since the idea of a tipping point is that it begins a chain of events that ends in severe deprivation, one complexity is that the same decision might constitute a negative tipping point for one person, and a minor setback for another. This raises the question of whether trying to intervene at ‘tipping points’ would constitute an excessive deprivation of freedom. To give an example, the same level of alcohol consumption may constitute the beginning of addiction for one person, and a non-ideal but ultimately sustainable level for another.

One way forward is to focus first and foremost on generic tipping points—vulnerabilities that are widespread and well-known within societies. For instance, continued stress exposure due to racist discrimination (Gee *et al*., 2019), or the severe insecurities that come with transitional phases in life, such as leaving institutionalised care facilities or secure workplaces, or becoming disabled, are potential negative health-related tipping points for many. There is some evidence, for instance, that many rough sleepers in Global North countries are veterans or prison leavers (Tsai, 2018; Bozkina and Hardwick, 2021; Wood *et al.*, 2022; European Observatory on Homelessness, 2023). Sufficientarian health capital justice aims at balancing common health threats that make people significantly vulnerable through diverse support measures. We cannot discuss, at this point, which measures would be justified to prevent negative tipping points. There are, of course, already established policies, for instance, to build a network of aftercare social support (see, e.g. National Guideline Alliance (UK), 2022), which would need to be assessed from the perspective of sufficientarian justice.

Yet even if we assume that a specific tipping point represents an important threshold, this does not mean that a sufficientarian approach to health capital must endorse the state or its institutions stepping in heavy-handedly every time someone might be approaching a tipping point. Rather, we suggest that a graded approach is more appropriate. Where people approach a potential tipping point—judged by reference to where others have made similar choices and knowledge about the individual—this warrants some level of intervention, such as engaging with the individual about their behaviours, and offering advice and support. In some such cases, no depletion cycle will occur; the individual may change their own behaviour (perhaps with help from friends and family), or the behaviour may simply be maintained at a non-optimal but reasonable level. In other cases, as it becomes clearer that an individual is being drawn into a depletion cycle, greater levels of intervention may be warranted, including monitoring of behaviour, correcting the damage done to the individual’s long-term health capital as a result of their choices, and potentially coercive interventions to avoid serious further damage.

The dynamic nature of health capital also tells us something about a question that has taken on increasing prominence in debates around justice, namely the timeframe across which justice applies. Many theories of justice take people’s whole lives as their basic unit; for instance, many egalitarians would have us aim for equality across people’s lives even if this meant considerable inequality at particular times. Dennis McKerlie (2013) challenges such an approach, insisting that we should also care about how well or badly off people are at particular times. And indeed, sufficientarians have been unusual among theories of justice in focussing on this latter question; often, though not always, ignoring the question of what might make for a sufficient life.

The idea of health capital is, we have suggested, an inherently dynamic one across time. In seeing health as a form of capital, and other goods as capital for health, it is natural to think about ideas that do not really make much sense except across some stretch of time. To ‘invest’ health capital for the future implies trading off goods now against goods later, while it only makes sense to think of ‘spending’ health capital in the present if one thinks about past investments or other kinds of accumulation. And this is, to some degree, in line with thinking about justice that conceives it as applying predominantly across people’s lifetimes (Rawls, 1971: 78; Dworkin, 1981: 304–5; Temkin, 1993; Wagland, 2012; Lippert-Rasmussen, 2015: 156; Bidadanure, 2016; Segall, 2016).

Importantly, we think that even if a person has had considerable amounts of healthy time in their life so far—and thus can be said to have been ‘wealthy’ in health capital at various stages—they will still have claims in the shorter term to help in maintaining their health capital. This might strike some as an odd perspective. For instance, Klemens Kappel and Peter Sandøe (1992: 314) also draw an analogy between health and capital, but they use it to justify giving lower priority for health care to older individuals. In their view, extending the life of someone who has already had a long life of good health is like ‘giving money to the rich rather than to the poor’. However, we suggest that a better analogy would be ‘giving money to someone who used to be rich’, but who has unavoidably spent much of their wealth so that they are now much poorer. That someone has enjoyed wealth across their life is clearly relevant to justice—if they have spent their money well, they have used it to acquire value that others did not have the chance to enjoy. But the fact that someone used to be wealthy does not exhaust their claim to help if they are now without resources. Similarly, the fact that someone has enjoyed good health capital across a reasonable length of life does not mean that their claims are exhausted.

## Objections

One might think that a sufficientarian view has an advantage over egalitarianism: After all, measuring equality of health capital would require a precise sense of how different aspects of capital interact with one another, whereas a sufficientarian view insists that enough health capital requires sufficiency in a number of different areas and that we do not need to *compare* these different areas in order to see whether someone has achieved sufficiency. For instance, someone who has good health stock but *no* knowledge of how to use and maintain it does not have sufficient health capital. No amount of one ingredient of health capital can make up for an insufficiency of another.

However, while we have indeed explored a sufficientarian approach to health capital, the complexity involved presents challenges for sufficientarianism (Wolff and de Shalit, 2007: Chapter 1). When we come to an ethical or political response to insufficiency, the apparent pragmatic advantage of sufficiency is significantly weakened. It is easy to say that justice requires everyone to have sufficient health capital and that this requires sufficiency in each of a set of not-fully-fungible components of health capital (health stock; knowledge about health; health-promoting social circumstances; etc.). But in reality, we must sometimes decide between promoting or helping individuals to achieve different components of health capital. In addition, some level of precision is required in deciding how far one type of health capital can compensate for another that cannot be improved. For instance, it is often not possible to improve an individual’s health stock. An individual whose immune system is compromised may not be able to benefit from vaccination as other people can. From a justice perspective, then, we need to decide the extent to which other components of health capital should be invested to compensate for this disadvantage. And there may be reasonable disagreement about this.

So, there is no straightforward pragmatic argument for a sufficientarian approach to health capital. Our argument presented here is not intended to be a comprehensive defence of sufficiency as the correct pattern of justice, nor a rebuttal of distributive egalitarianism. Rather, we think that there is something to be said for a sufficientarian approach to the idea of health capital, and in this paper our aim is to offer a rough outline of such an approach, and some initial motivation for it.

There are at least two further issues that might be raised. The first is that our approach excludes individuals who lack some internal capacities for making investment decisions, and who will lack these capacities no matter how much they are supported; for instance, individuals with some forms of significant cognitive disability. It is important not to overstate this case: many people with cognitive disabilities are capable of thinking about these questions, either alone or with support, and setting priorities for their lives. But some are not. For such individuals, the capacity to make decisions for oneself about health capital investment must be externalised: a sufficient level of health capital requires some other, sufficiently well-motivated individual who can make decisions, informed as far as possible by the incapacitated person’s preferences, on their behalf. While it is generally preferable to retain the capacity to make such decisions yourself, such proxy decision-making still has considerable value compared with other alternatives, such as lacking even proxy control over one’s health capital (cf. Francis and Silvers, 2007; Kittay, 2019).

A second issue is whether what we have appealed to really supports a sufficientarian view. After all, some might argue that what matters is not that people have some bare minimum of capacity to protect health capital, but rather that they should have *equal* capacity. In our view, though, this is too invasive and fails to sufficiently respect people’s choices, at least at a practical level of operating a social system. People cannot be protected from every cost they might incur without excessively interfering in their investment decisions.

Luck egalitarians may insist that where people genuinely choose to make suboptimal health capital investments, there is no reason to compensate or prevent them from doing so, but that our framework should still be an egalitarian one, ensuring that people have equal health capital except where inequalities result from free choice. However, as Davies (2022) argues, such a view has unattractive implications when we consider a dynamic, iterative process such as health capital investment. Assume (implausibly) that we start from a position of complete health capital equality, and that some individuals make free choices that drive down their health capital holdings. Since this is a free choice, the luck egalitarian stance implies that we should allow these differences to stand. But now, due to the dynamic nature of health capital, those with lower health capital holdings will be more likely to make future decisions that further deplete their health capital, and so on. One way to resist this is to insist on a sufficientarian lower limit, below which people should not be allowed to fall even if that results only from a series of free choices. Luck egalitarians seem stuck insisting either that we must maintain strict equality, or that there is no principled point at which to intervene.

## Conclusion

In this paper, we have outlined a novel framework for theorising health-related social justice. We have argued that health capital provides a valuable conceptual tool to normatively assess social and individual conditions. Health capital comprises elements of organismic health and the means of health production. It is hence an inclusive framework that allows expansion in numerous dimensions. For example, it is well suited to address concerns of health justice over time. It is specifically concerned with health-related vulnerabilities and threats, which makes it less demanding than theories that aim at equality in a specific respect. Our focal point is still strongly connected to the traditional aim of health care to deal with instances of disease. Securing minimal health is also our aim, although the relevant means are much broader than the provision of medical resources. Here we are inspired by approaches to health justice found in the public health literature. At the same time, our framework can prevent some of the pitfalls hampering related debates on health justice and the social determinants of health. In terms of social justice our focus in this paper has been on tipping points of health capital depletion, which can incapacitate people to maintain their health. Justice requires that everybody has enough to maintain their health over time. This calls for a sufficientarian account of health capital justice. We have only laid the foundation for such a theory. Finally, we have hinted at some possible normative implications of our approach, including possible assessments of public policies. Further work and critical engagement will establish the full potential of our framework.

## Footnotes

1 The broad conception of health capabilities leads to further awkward results, for instance the inclusion of the ‘capability of a person to look more muscular’ (Prah Ruger, 2010, 93), which is then qualified by calling it a ‘non-central’ health capability (see also ibid. 76f.). We believe that it is more plausible to consider which organismic conditions and external means are required to maintain health in the sense of balancing the threats of disease.

2 Arrow and his co-authors (2014, 131) discuss *the value of* health capital as the summation of three components: direct wellbeing, productivity and longevity. Schneider-Kamp (2021, 212) defines ‘health capital as the aggregate of the actual or potential resources possessed by a given agent that have the capacity to affect the position of agents in the social field of health’. Her definition is influenced by Pierre Bourdieu’s seminal work on cultural capital. Nikolas Rose uses the term *biocapital* (Rose 2007, 19, 255ff.), mainly to refer to the commodification of ‘life itself’, but he does not provide a definition.
